# Neonaucline, a New Indole Alkaloid from the Leaves of *Ochreinauclea maingayii* (Hook. f.) Ridsd.(Rubiaceae)

**DOI:** 10.3390/molecules17010267

**Published:** 2011-12-28

**Authors:** Mat Ropi Mukhtar, Norfaizah Osman, Khalijah Awang, Hazrina Hazni, Ahmad Kaleem Qureshi, A. Hamid A. Hadi, Kazuma Zaima, Hiroshi Morita, Marc Litaudon

**Affiliations:** 1 Department of Chemistry, Faculty of Science, University of Malaya, 50603 Kuala Lumpur, Malaysia; Email: faizah05@gmail.com (N.O.); khalijah@um.edu.my (K.A.); hazrinahazni@um.edu.my (H.H.); ahamid@um.edu.my (A.H.A.H.); 2 Faculty of Pharmaceutical Sciences, Hoshi University, Shinagawa-ku, Tokyo 142-8501, Japan; Email: moritah@hoshi.ac.jp (H.M.); 3 Institut de Chimie de la Substances Naturelles, Centre Nationale de la Recherches Scientifique, 91198, Gif-sur Yvette, Cedex, France; Email: marc.litaudon@icsn.cnrs-gif.fr (M.L.)

**Keywords:** *Ochreinauclea maingayii*, neonaucline, nauledine, cadamine, Rubiaceae, alkaloid

## Abstract

A new indole alkaloid; neonaucline (**1**), along with six known compounds–Cadamine (**2**), naucledine (**3**), harmane, benzamide, cinnamide and blumenol A–were isolated from the leaves of *Ochreinauclea maingayii* (Rubiaceae). In addition to that of compound **1**, ^13^C-NMR data of cadamine (**2**) and naucledine (**3**) were also reported. Structural elucidations of these alkaloids were performed using spectroscopic methods especially 1D- and 2D-NMR, IR, UV and LCMS-IT-TOF. The excellent vasorelaxant activity on isolated rat aorta was observed for the alkaloids **1**–**3** after injection of each sample at 1 × 10^−5^ M.

## 1. Introduction

*Ochreinauclea maingayii* (Hook. f.) Ridsd. (Rubiaceae) is a medium size tree distributed in Peninsular Malaysia, Borneo, Sumatra and Thailand [1,2,3,4]. The timber of *Ochreinauclea* species shares the standard Malaysian name “*bangkal*” or “mengkal” with *Nauclea* and *Neonauclea* species [5]. There has been no report of other phytochemical study and medicinal value of *Ochreinauclea maingayii* so far. In continuation of our research on plants from the Rubiaceae family [6], we have embarked a study on the CH_2_Cl_2_ extract of the plant *Ochreinauclea maingayii*. The present study has led to the isolation of a new indole alkaloid, neonaucline (**1**) together with cadamine (**2**) [7,8,9], naucledine (**3**) [10,11,12,13], harmane [14,15], blumenol A [16], bezamide, and cinnamide.

**Figure 1:**
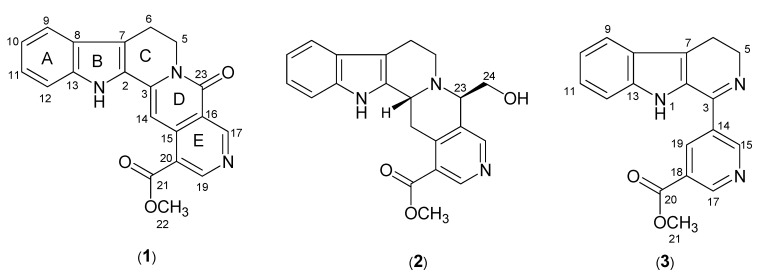
Structures of neonaucline (**1**), cadamine (**2**), and naucledine (**3**).

## 2. Results and Discussion

**Figure 2:**
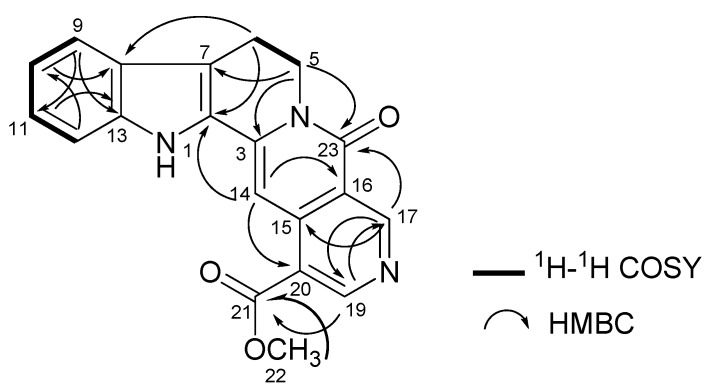
Selected 2D NMR correlations of neonaucline (**1**).

Neonaucline (**1**) was isolated as a yellowish amorphous solid. The LCMS-IT-TOF spectrum of **1** showed a pseudomolecular ion peak, [M+H] at *m/z* 346.1140, corresponding to the molecular formula of C_20_H_16_N_3_O_3_. The IR spectrum revealed absorption bands at 3,364 and 1,731 cm^−1^ for the stretching vibrations of –NH and –CO groups respectively. In the ^1^H-NMR spectrum, signals for seven aromatic protons due to one methoxy singlet and one –CH_2_–CH_2_–N– group were observed, thus suggesting an indolopyridinequinolizinone type of skeleton [17]. Among the seven aromatic proton signals, two resonated as doublet of doublets (dd) at δ 7.35 and 7.18 (H-10 and H11), two doublets at δ 7.64 and 7.46 (H-9 and H-12), and three singlets at δ 7.88, 9.32 and 9.69 assignable to H-14, H-17, and H-19, respectively. Further analysis of the ^1^H-NMR and ^13^C-NMR spectra showed that **1** is very similar to naucletine [18] except that the former revealed the presence of a singlet representing a methoxy group at δ_H_ 4.00 and δ_C_ 52.5. The position of COOMe attached to C-20 (δ 120.4) in ring E was confirmed based on the HMBC correlations of H-14/C-20 (δ 120.4), H-19/C-21 (δ 166.4), H-22/C-21 and H-17/C-23 (δ 166.4), respectively as shown in Figure 2. The ^13^C-NMR spectrum revealed 20 carbon signals due to eight quaternary carbons, seven methines, two methylenes, one methoxy group and two carbonyl groups. The ^1^H-NMR (400 MHz) and ^13^C-NMR (100 MHz) spectral assignments performed by extensive 2D-NMR experiments (COSY, HSQC and HMBC) were summarized in Figure 2 and Table 1.

**Table 1:** ^1^H-NMR (400 Hz) and ^13^C-NMR (100 Hz) spectral data of neonaucline (**1**) and cadamine (**2**) in CDCl_3_.

Position	^1^H (δ_H_, Hz) (1)	^13^C (δ_C_, CDCl_3_)	^1^H (δ_H_, Hz) (2)	^13^C(δ_C_,CDCl_3_)
N-H	8.72s	-	7.95s	-
2	-	127.4	-	134.9
3	-	138.2	4.43m	46.3
5	4.54t (2H,6.8)	40.7	2.94, 3.22m	47.6
6	3.19t (2H,6.8)	19.4	2.80, 2.98m	21.9
7	-	116.9	-	107.9
8	-	125.7	-	126.9
9	7.64d (7.8)	119.9	7.48 d (8.0)	118.3
10	7.18dd (7.8,7.8)	120.9	7.10 dd (8.0,8.0)	119.4
11	7.35dd (7.8,7.8)	125.6	7.16 dd (8.0,8.0)	121.9
12	7.46d (7.8)	111.9	7.27 d (8.0)	111.1
13	-	138.6	-	136.2
14	7.88 s	95.1	3.25,3.51 m	28.3
15	-	141.9	-	124.5
16	-	117.8	-	130.8
17	9.32 s	155.4	8.51 s	151.8
19	9.69 s	154.2	8.96 s	150.0
20		120.4	-	144.8
21	-	166.4	-	166.3
22-OMe	4.00 s	52.5	3.88 s	52.4
23	-	166.4	4.10 m	62.6
24	-	-	3.61, 3.75 m	63.6

Cadamine (**2**) and naucledine (**3**) were isolated as a reddish and yellowish amorphous solid. The LCMS-IT-TOFF spectra showed pseudomolecular ion peaks, [M+H]^+^ at *m/z* 364.1638 [C_21_H_21_N_3_O_3_] and *m/z* 306.1213 [C_18_H_15_N_3_O_3_], respectively. The ^1^H-NMR data of cadamine (**2**) was reported previously based on cadamine acetate [7] whereas **3** were first isolated from *Nauclea diderrichi* [10]. We herein report the ^13^C-NMR data for both compounds which has not been reported yet [7,8,9,10,11,12,13]. In view of that, complete assignments were established through various NMR measurements; DEPT, HMQC, HMBC and NOESY spectra. The ^13^C-NMR spectra of cadamine (**2**) and naucledine (**3**) indicated the presence of 21 and 18 carbons, respectively, as shown in Table 1 and Table 2.

**Table 2:** ^1^H-NMR (in DMSO-d6 and CDCl_3_) and ^13^C-NMR (100 Hz, CDCl_3_) spectral data of naucledine (**3**).

Position	*^1^H (τ, DMSO-*d_6_*)	^1^H (δ_H_, Hz)	^13^C (δ_C_,)
N-H	0.90s	8.35 s	
2	-	-	126.3
3	-	-	156.3
5	6.36m	4.13 (t, 6.8)	49.3
6	6.96m	3.03 (t, 6.8)	19.3
7	-	-	
8	-	-	125.5
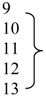		7.68 (d, 8.3)	120.3
	2.2-3.1	7.23 (dd, 7.8, 8.3)	120.9
	(3peaks)	7.33 (dd, 7.8,8.3)	125.4
		7.42 (d, 8.3)	112.4
	-	-	136.9
14	-	-	125.5
15	0.65d	9.30 (d, 1.96)	151.8
17	0.73d	9.18 (d, 1.96)	152.6
18	-	-	133.3
19	0.88t	8.68 (t, 2.20, 1.96)	136.5
20			165.5
21	6.08s(3H)	3.98 (s)	52.8

* ^1^H-NMR data is reproduced from Murray *et al*. [11].

**Figure 3:**
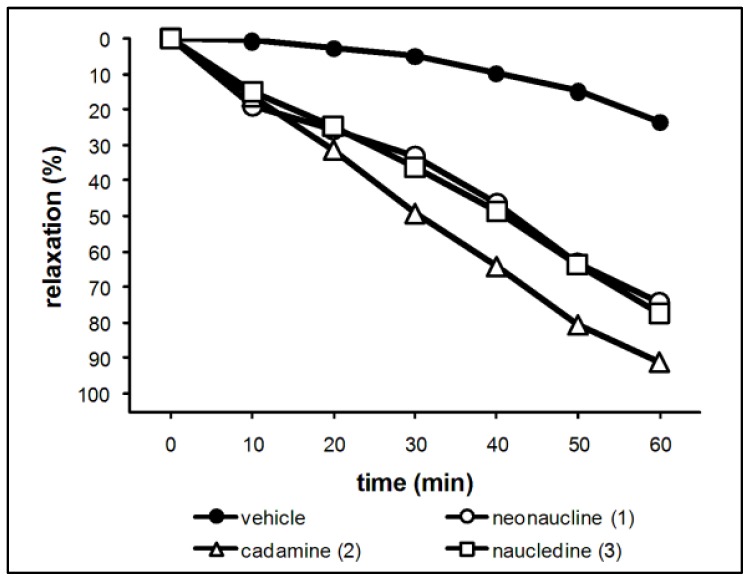
Vasorelaxant effects of neonaucline (**1**; 10^−^^5^M), cadamine (**2**; 10^−^^5^M), and naucledine (**3**; 10^−^^5^M) on endothelium-intact rings cut from rat arteries pre-contracted with PE (0.3 μM).

Vasodilators are useful for treatment of cerebral vasospasm and hypertension, and for improvement of peripheral circulation [19]. When phenylephrine (PE) 3 × 10^−7^ M was applied to thoracic aortic rings with endothelium after achieving a maximal response, we added neonaucline (**1**; 1 × 10^−5^M), cadamine (**2**; 1 × 10^−5^M), and naucledine (**3**; 1 × 10^−5^M). The excellent activity could be observed for these three alkaloids (**1**–**3**) after injection of each sample at 1 × 10^−5^M as shown in Figure 3.

In the previous papers, we have reported vasorelaxant activities of some bisbenzylisoquinoline alkaloids such as α'-oxoperakensimines A–C from *Alseodaphne perakensis* and *A. corneri* [20,21], and *N*-allyllaurolitsine from *Litsea lancifolia* [22]. These vasorelaxant effects may be mediated through the increased release of NO from endothelial cells, inhibition of calcium influx from extracellular space through voltage-dependent calcium channels (VDC) and/or receptor-operated Ca^2+^-channels (ROC), and also through the increased release of NO from endotheliul cells and opening of voltage-gated K^+^-channels. The mode of action of these alkaloids on vasorelaxant activity is under investigation.

## 3. Experimental

### General

Spectra were recorded on the following instruments: UV, Shimadzu UV-160A UV-Visible spectrophotometer; IR, Perkin Elmer 1600; NMR, JEOL ECA 400 MHz; LCMS-IT-TOF, Shimadzu. All solvents, except those used for bulk extraction are AR grade. Silica gel 60 F_254_ was used for column chromatography. Glass and aluminium supported silica gel 60 F_254_ plates were used for preparative TLC. TLC sports were visualized under UV light (254 and 365 nm) followed by spraying with Dragendorff’s reagent for alkaloid detection. 

*Plant Material*: The leaves of *Ochreinauclea maingayii* were collected at Reserve Forest Sg. Tekam, Jerantut, Pahang, Malaysia, in 17 February 2009. The plant species was identified by Prof. Colin E. Ridsdale from Leiden University, Netherland. A voucher specimen (KL5625) was deposited in the Herbarium of Department of Chemistry, University of Malaya, Malaysia. 

*Extraction and the isolation*: A total of 2.0 kg of dried and grounded leaves of *Ochreinauclea maingayii* was extracted with CH_2_Cl_2_. Extraction of alkaloids was carried out in the usual manner, which has been described in detail [17,18,19]. Finally, the extract was concentrated to give crude alkaloids of 3.0 g in weight. The isolation and purification of compounds **1**–**3** by a small column chromatography (column dimension = 1.0 cm, length = 25 cm, silica gel 60, 70–230 mesh ASTM; Merck 7734) and preparative TLC (PTLC Merck KGaA silica gel 60 F254) yielded 0.13% of neonaucline (**1**), (CH_2_Cl_2_-MeOH; 98:2), cadamine (**2**), (0.76%, CH_2_Cl_2_-MeOH; 96:4) and naucledine (**3**), (0.08%, CH_2_Cl_2_-MeOH; 96:4), respectively.

*Vasodilation Assay*: The vasorelaxant activities of all the alkaloids **1**–**3** were tested using the same procedure as reported previously by Morita *et al.* [20]. The animal experimental studies were conducted in accordance with the Guiding Principles for the Care and Use of Laboratory Animals, Hoshi University and under the supervision of the Committee on Animal Research of Hoshi University, which is accredited by the Ministry of Education, Science, Sports Culture, and Technology of Japan.

*Neonaucline* (**1**). Yellowish amorphous solid, LCMS-IT-TOFF at *m/z* 346.1140 ([M+H]^+^; calcd for C_20_H_16_N_3_O_3, _346.1192); UV (MeOH) 376, 284, 204nm; IR (KBr) λ_max_ 3,364, 1,737 and 1,731 cm^−1^; ^1^H and ^13^C-NMR: see Table 1.

## 4. Conclusions

**Scheme 1:**
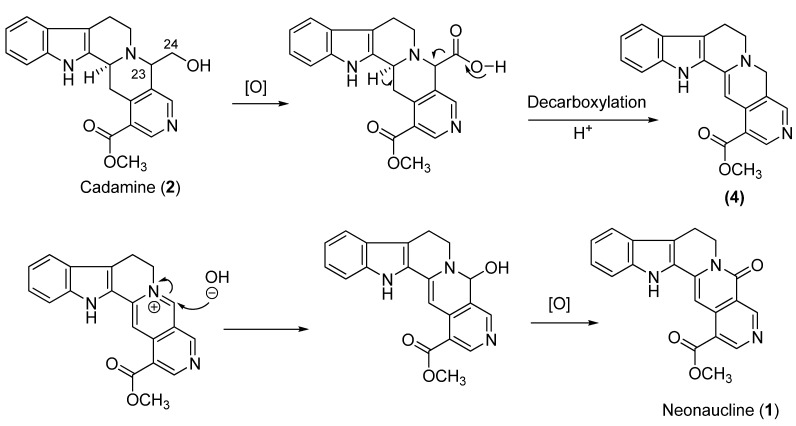
Biogenetic pathway for neonaucline (**1**).

This is the first report on the phytochemical and biological studies on the species of *Ochreinauclea maingayii*. One new indole alkaloid- neonaucline (**1**) along with six known compounds- three alkaloids (cadamine **2**, naucledine **3** and harmane), one nor-isoprenoid-type (blumenol A), bezamide and cinnamide were isolated from the leaves of this species. The proposed biogenesis of **1** from cadamine (**2**) is illustrated in Scheme 1. The latter is oxidized at C-24 followed by decarboxilation and protonation of the carbanion to give compound **4**. Then, **4** will be hydroxylated at C-23 followed by oxidation to give neonaucline (**1**). The excellent vasorelaxant activity on isolated rat aorta was observed for the alkaloids **1**–**3** after injection of each sample at 1 × 10^−5^ M.
